# HSP47 at the Crossroads of Thrombosis and Collagen Dynamics: Unlocking Therapeutic Horizons and Debates

**DOI:** 10.1055/a-2599-4925

**Published:** 2025-06-05

**Authors:** David M. Smadja, Alberto F. Chocron, M. Marc Abreu

**Affiliations:** 1 Department of Hematology, Assistance Publique – Hôpitaux de Paris, Georges Pompidou European Hospital, Paris, France; 2 Université Paris Cité, Institut National de la Santé et de la Recherche Médicale, Paris Cardiovascular Research Center, Paris, France; 3 Research Service, Miami Veteran Administration Medical Center, Miami, Florida, United States; 4 Department of Medicine, BTT Medical Institute, Aventura, Florida, United States; 5 Department of Engineering, BTT Medical Institute, Aventura, Florida, United States

**Keywords:** heat shock protein 47, collagen, thrombosis, angiogenesis, cancer therapy, hyperthermia

## Abstract

Heat shock protein 47 (HSP47), a collagen-specific molecular chaperone encoded by the *SERPINH1* gene, has emerged as a groundbreaking focus in thrombosis research. Recent findings published in “Science” have revolutionized our understanding of thrombosis, identifying HSP47 as a critical mediator in a new thrombosis target for treatment. This discovery not only unveils a novel pathway in thrombosis but also opens new avenues for therapeutic intervention. HSP47's significance extends beyond thrombosis, influencing pathological processes such as fibrosis and cancer. In fibrosis, its upregulation promotes collagen deposition, while its dysregulation in osteogenesis imperfecta (OI) Type X underscores the protein's indispensable role in collagen biosynthesis. The therapeutic challenge lies in balancing HSP47 inhibition to reduce fibrotic burden without impairing its essential physiological functions. In cancer, HSP47 plays dual roles. It supports tumor progression through collagen stabilization and metastasis facilitation while contributing to tissue repair under hyperthermia treatment combined with radiotherapy or chemotherapy. However, its overexpression can exacerbate tumor aggressiveness via mechanisms such as angiogenesis and epithelial–mesenchymal transition.

This review emphasizes the pivotal discovery of HSP47's thrombogenic role and its broader implications in disease biology. These findings mark a paradigm shift in thrombosis research and underscore the potential of HSP47 as a target in diverse pathological contexts, from platelet-driven diseases to fibrotic and oncological disorders.

## Introduction

**Fig. 1:**
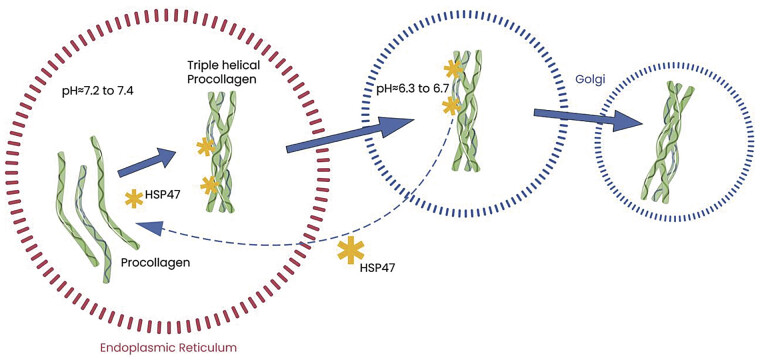
Mechanism of procollagen folding and stabilization mediated by HSP47. Procollagen enters the endoplasmic reticulum (ER), where HSP47 binds to stabilize its triple-helical structure. As procollagen traffics through the Golgi, the progressive acidification of the organelle, from approximately 7.2 to 7.4 in the ER to 6.3 to 6.7 in the cis-Golgi, facilitates the dissociation of HSP47. This pH-dependent release is crucial for proper collagen export. HSP47 is then recycled back to the ER via COPI-mediated retrograde transport. HSP47, heat shock protein 47.

Type I collagen serves as the primary structural component of the extracellular matrix (ECM) and plays a critical role in fibrosis and platelet adhesion. 1 Structurally, type I collagen comprises two α1 chains and a single α2 chain, which assemble into a triple-helical configuration within the endoplasmic reticulum (ER) before being transported to the Golgi apparatus. 2 This collagen is inherently thermally unstable at physiological temperatures, necessitating the assistance of specialized chaperone proteins to ensure proper folding and stability. 3 One such indispensable chaperone is heat shock protein (HSP) 47 (HSP47), a collagen-specific binding protein encoded by the *SERPINH1* gene and residing in the ER. 4 HSP47 is structurally related to the serpin family but does not function as a serine protease inhibitor. 5 6 Unlike other chaperones such as HSP60, HSP70, and HSP90, which interact with a wide range of substrates, 7 8 HSP47 exhibits unique specificity, binding exclusively to procollagen. HSP47 is crucial for the proper folding of procollagen within this compartment. Indeed, this ER-resident molecular chaperone binds to the Gly-Xaa-Arg motif in procollagen, stabilizing the triple helix. 5 6 By preventing local unfolding and aggregation, HSP47 enables the successful formation of the collagen's helical structure ( Fig. 1 ). 5 6 The acidification of organelles, particularly the ER and Golgi apparatus, plays a crucial role in the function of HSPs. HSP47 relies on the pH gradient within these organelles to regulate collagen maturation and secretion. However, its dissociation from procollagen is pH-dependent, occurring in the acidic environment of the cis-Golgi. In the ER, which maintains a neutral pH of approximately 7.2 to 7.4, HSP47 binds newly synthesized procollagen and prevents premature aggregation. This binding is critical for the correct folding and stabilization of procollagen. As procollagen is transported to the Golgi, the pH progressively decreases, reaching approximately 6.3 to 6.7 in the cis-Golgi. This acidification triggers the release of HSP47 from the collagen molecule. 5 9 The dissociation of HSP47 in this environment is essential for the proper trafficking of procollagen to the ECM. If the acidification process is disrupted, such as through the inhibition of vacuolar H ^+^ -ATPase by compounds like bafilomycin A1, HSP47 remains bound to procollagen, leading to its retention and impaired secretion. In the trans-Golgi, where the pH is further reduced to around 6.0, HSP47 is recycled back to the ER via coat protein complex I-mediated retrograde transport. This recycling process ensures that HSP47 is available to assist in the maturation of newly synthesized procollagen molecules. Acidification within organelles is essential for the function of various HSPs involved in protein maturation, trafficking, and degradation. Other HSP families, such as HSP70 and HSP90, rely on pH-sensitive mechanisms to regulate protein folding and release in the ER, protein degradation in lysosomes, and the activation of stress responses in acidic microenvironments.

HSP47 involvement in both physiological and pathological processes is now well established. 10 Its involvement in collagen synthesis and maturation has many implications for diseases where collagen integrity is compromised, such as OI, fibrosis, and certain cancers. 11 Recent studies have expanded our understanding of HSP47's functions, suggesting that it also plays significant roles in thrombosis or angiogenesis. In thrombosis, HSP47 is found on activated platelets, where it supports platelet aggregation by enhancing collagen interactions and thrombus formation. 12 13 This association with thrombosis, particularly in conditions involving immobilization, has spurred interest in HSP47 as a target for thromboprotective therapies, as demonstrated in studies involving hibernating animals, 14 which exhibit natural resistance to clot formation. However, targeting HSP47 for thrombosis prevention or other therapeutic applications, like idiopathic pulmonary fibrosis (IPF), must take into account its essential role in collagen stability, as deficiency or inhibition of HSP47 can lead to severe skeletal defects, as observed in OI Type X. 15

This review aims to consolidate current knowledge on HSP47's diverse roles, exploring its implications in collagen biology, thrombosis, angiogenesis, but also questions about the relevance of HSP47 target in fibrosis and cancer. By addressing both the therapeutic promise and the challenges of targeting HSP47, we hope to elucidate its potential as a multifaceted biomarker and therapeutic target across a spectrum of diseases, emphasizing the importance of context in understanding HSP47's complex biology.

### HSP47 as a Target in Pulmonary Fibrosis and Cancer

Many diseases are associated with either collagen accumulation or malfunction. 16 Fibrosis is one such condition, particularly when abnormal collagen accumulates in organs such as the liver, 17 18 lungs, 19 and kidneys. 20 21 We will focus in the next paragraph on pulmonary fibrosis. Indeed, HSP47 expression is upregulated by key profibrotic factors in pulmonary fibrosis models. 22 TGF-β1 enhances HSP47 and collagen type I expression in normal human fibroblasts and alveolar epithelial cells. 23 Similarly, IL-1β promote HSP47 synthesis. 24 In bleomycin-induced pulmonary fibrosis, HSP47 expression is elevated in myofibroblasts, alveolar epithelial cells, and macrophages, correlating with increased collagen deposition. 25 26 Aging amplifies this response, with older mice showing greater HSP47 and collagen levels. 27 Exposure to TGF-β1, a key extracellular regulator of fibrosis, induced an upregulation in both HSP47 and collagen type I at the mRNA and protein levels in normal human lung fibroblasts and alveolar epithelial cells. 28 29 ER stress and unfolded protein response pathways further regulate HSP47, as demonstrated by increased expression in fibrotic lungs. 30 Conversely, HSP47 levels decrease in conditions like acute respiratory distress syndrome associated with diffuse alveolar damage. 31 Finally, HSP47 expression is significantly higher in fibrotic lungs of patients with IPF and correlates with disease severity. 11 25 32 33 It is localized to myofibroblasts and type II alveolar epithelial cells in areas of active fibrosis. 34 Serum HSP47 levels are elevated in acute IPF exacerbations and acute interstitial pneumonia, making it a potential biomarker. 11 Additionally, anti-HSP47 autoantibodies are prevalent in autoimmune and fibrotic diseases, likely triggered by tissue damage and inflammation. 35 Given HSP47's central role in fibrosis, therapeutic strategies aimed at modulating HSP47 expression and downstream effects may help mitigate fibrotic diseases. Nintedanib, an already approved treatment for IPF and other progressive fibrosing interstitial lung diseases, has been shown to suppress lung fibroblast proliferation, motility, and myofibroblast differentiation. 36 37 38 39 40 41 42 It also reduces TGF-β1-induced collagen production but has minimal effect on HSP47 protein levels, despite lowering its mRNA through *SERPINH1* suppression. The other validated treatment for IPF, pirfenidone, is an orally administered synthetic molecule that slows disease progression and reduces fibrosis in organs such as the kidney, liver, and heart. 43 It achieves this by inhibiting profibrotic mediators such as TGF-β1, TNF-α, and IL-1β, while also exhibiting anti-inflammatory and antioxidant properties. Notably, pirfenidone decreases HSP47 and collagen expression in lung cells and reduces the presence of HSP47-positive fibroblasts in fibrotic tissues. 28 29 41 A variety of drugs have demonstrated the ability to influence HSP47, shedding light on its potential as a therapeutic target in fibrosis. Diallyl sulfide, a sulfur compound derived from garlic, has shown significant antifibrotic effects by suppressing both HSP47 and α-smooth muscle actin expression in models of lung fibrosis. 44 Aminoguanidine, known for its ability to inhibit advanced glycation end-products linked to inflammation and fibrosis, markedly reduces HSP47 and collagen expression in fibrotic tissues. 45 46 Corticosteroids like budesonide and fluticasone are also effective, downregulating *SERPINH1* mRNA and collagen production in lung fibroblasts, making them relevant in treating fibrotic conditions. 47 Moreover, prieurianin and its analogs, such as dregeanin—naturally occurring limonoids from plants like *Trichilia prieuriana* and *Aphanamixis polystachya* —exhibit diverse biological properties, including antifibrotic activity, by targeting HSP47 at the protein–collagen interface. 48 Specific compounds designed to target the pH-dependent dissociation of Hsp47 from procollagen in the Golgi are still under active investigation. However, several general approaches to modulating Hsp47 function and its interaction with procollagen have been explored. Glycine analogs, such as Gly-Pro-Arg, mimic collagen sequences and have shown potential to interfere with Hsp47 binding, which could indirectly affect its dissociation from procollagen. Agents that alter the pH gradient in the Golgi, including ammonium chloride and bafilomycin A1 (a vacuolar H ^+^ -ATPase inhibitor), can disrupt the acidic conditions required for Hsp47 release, thus influencing its activity. 49 50 Adding to these strategies, SERPINH1 siRNA has emerged as a promising approach, effectively silencing HSP47 expression in animal models, reducing fibrosis markers, and improving lung function. 51 Collectively, these therapeutic interventions emphasize the critical role of HSP47 in fibrosis and its value as a focal point for developing treatments to enhance patient outcomes ( Table 1 ) 5 41 44 45 47 48 49 51 52 53 and across multiple fibrotic diseases (e.g., pulmonary, liver, kidney, or skin fibrosis; Table 2 ). 54 55 56 57 58 59 60 61 62 63 64 65

**Table 1:** Summary of therapeutic agents affecting HSP47 expression or function across fibrotic and inflammatory diseases

Drug/Compound	Target disease/condition	Effect on HSP47	References
Nintedanib	Idiopathic pulmonary fibrosis	Reduces SERPINH1 mRNA (HSP47 precursor) but minimal effect on HSP47 protein levels	Knüppel et al. (2017) 41
Pirfenidone	IPF, fibrotic diseases	Decreases HSP47 and collagen expression in lung cells, reduces HSP47-positive fibroblasts—reduces SERPINH1 mRNA (HSP47 precursor) but minimal effect on HSP47 protein levels	Knüppel et al. (2017) 41
Diallylsulfide	Lung fibrosis	Suppresses HSP47 and α-SMA expression	Kalayarasan et al. (2013) 114
Aminoguanidine	Fibrosis, inflammation	Reduces HSP47 and collagen expression	Chen et al. (2009) 45
Corticosteroids (budesonide, fluticasone)	Fibrosis, lung diseases	Downregulate SERPINH1 mRNA, decrease collagen production	Goulet et al. (2007) 47
Prieurianin and Dregeanin	Fibrosis	Targets HSP47 at protein–collagen interface	Vergoten et al. (2024) 48
Bafilomycin A1	Various diseases	Disrupts acidic conditions required for HSP47 release from procollagen	Satoh et al. (1996) 49
SERPINH1 siRNA	Fibrosis (lung, liver, kidney)	Suppresses HSP47 expression, reduces fibrosis markers	Li et al. (2021) 115
Epigallocatechin gallate	?	?	Okuno et al. (2021) 52
Col003	Ischemic stroke and fibrosis	Blocking interactions between collagen and HSP47	Ito et al. (2017), 6 Wu et al. (2022) 53
Toolkit peptides	?	Peptides blocking interactions between collagen and HSP47	Cai et al. (2021) 84

Abbreviations: HSP47, heat shock protein 47; IPF, idiopathic pulmonary fibrosis.

This table presents an overview of key compounds and drug candidates investigated for their effects on HSP47 in the context of fibrosis, inflammation, and thromboinflammatory disorders. The agents listed—ranging from U.S. Food and Drug Administration-approved drugs (e.g., nintedanib, pirfenidone) to experimental peptides—target various stages of HSP47 regulation, including transcription (SERPINH1 mRNA), translation, and protein–collagen interaction. Mechanisms include suppression of collagen synthesis, disruption of HSP47–collagen binding, and inhibition of fibroblast activation. The table also highlights the variability in therapeutic impact, including instances of incomplete protein-level inhibition despite reduced mRNA levels.

**Table 2:** Therapeutic effects of in vivo HSP47 inhibition across multiple fibrotic disease models

Disease	Model	Organ	Therapeutic outcome	References
Scleroderma	Subcutaneous implantation of keloid in mice	Skin	Reduced collagen—reduced keloid volume	Chen et al. (2011) 54
Pulmonary fibrosis	Oral administration of paraquat (20 mg/kg/day) in rats	Lung	Reduction in pulmonary fibrosis with reduced HSP47 and collagen	Hagiwara et al. (2007) 55
Pulmonary fibrosis	Intratracheal administration of bleomycin in rats	Lung	Reduced HSP47 and collagen expression—improved survival	Hagiwara et al. (2007) 56
Liver fibrosis	Percutaneous instillation of japonicum cercariae	Liver	Improved survival, reduced liver markers and fibrosis	Huang et al. (2014) 57
Scleroderma	Intradermal administration of Bleomycin in mice	Skin	Reduced NOX4, α-SMA, collagen type I—reduced skin thickness	Morry et al. (2015) 58
Kidney fibrosis	Unilateral ureteral obstruction in mice	Kidney	Reduced collagens I, II, IV—decreased macrophage infiltration	Xia et al. (2008) 59
Peritoneal fibrosis	Intraperitoneal injection of chlorhexidine gluconate	Peritoneum	Suppressed collagen and macrophage infiltration	Obata et al. (2012) 60
Liver cirrhosis	Intraperitoneal injection of dimethylnitrosamine in rats	Liver	Prolonged survival—reduced collagen and fibrosis	Sato et al. (2008) 61
Pancreatic fibrosis	Jugular injection of dibutyltin dichloride	Pancreas	Reduced collagen and fibrosis	Ishiwatari et al. (2013) 62
Pulmonary fibrosis	Intratracheal administration of bleomycin	Lung	Reduced collagen and cytokines—improved lung structure	Otsuka et al. (2017) 63
Dry eye	Mouse model of GVHD	Eye	Reduced HSP47—restored tear secretion—reduced fibrosis	Ohigashi et al. (2019) 64
Skin fibrosis	Cutaneous chronic GVHD in mice	Skin	Targeting HSP47+ myofibroblasts without inducing immunosuppression	Yamakawa et al. (2018) 65

Abbreviation: HSP47, heat shock protein 47; GVHD, graft-versus-host disease.

This table summarizes preclinical studies evaluating the outcomes of targeting HSP47 in various fibrotic diseases, including pulmonary, liver, kidney, pancreatic, and skin fibrosis. HSP47 inhibition was achieved through diverse models involving bleomycin, paraquat, apioncin, or chemical inducers. These studies support the therapeutic relevance of HSP47 modulation but also underscore the need for controlled targeting to prevent excessive tissue remodeling or impairment of physiological repair responses.

Fibrosis and cancer share common features, particularly in their reliance on collagen remodeling and cellular proliferation to drive disease progression. In both conditions, excessive collagen deposition alters the structure and function of the ECM, leading to tissue stiffness and disruption of normal cellular environments. HSP47 is overexpressed in various cancers, 66 67 68 where its upregulation enhances tumor aggressiveness, though the underlying mechanisms remain unclear. The *SERPINH1* gene, which encodes the heat-inducible protein HSP47, is situated on chromosome 11q13.5, a genomic hotspot commonly amplified in various human cancers. 69 Moreover, data from the University of ALabama at Birmingham CANcer (UALCAN) cancer database reveal that HSP47 mRNA levels are significantly elevated in multiple tumor types, including bladder, breast, colon, liver, lung, and stomach cancers, compared with normal tissues. Kaplan–Meier survival analysis further shows that higher HSP47 expression is strongly associated with poorer overall survival in patients with several cancers, such as bladder, liver, and lung adenocarcinomas. 70 Understanding these links is critical for advancing cancer treatment strategies. HSP47 plays a multifaceted role in cancer progression, influencing tumor growth, metastasis, and angiogenesis. 70 Overexpression of HSP47 promotes tumor cell proliferation by interacting with ER chaperones and stabilizing collagen production, essential for the ECM. 66 71 It also enhances epithelial–mesenchymal transition, a process critical for metastasis, by regulating key proteins in the Wnt/β-catenin and TGF-β pathways. 72 Additionally, HSP47 stabilizes Discoidin domain receptors (a class of collagen-binding receptor tyrosine kinases) DDR2, facilitating cancer cell migration and invasion. 73 In metastatic breast cancer, HSP47 strengthens actin filament contractility, enhancing the spread of cancer cells. 74 It also promotes cancer cell–platelet interactions, which protect circulating tumor cells and support their colonization in distant organs through the HSP47–collagen axis. 71 Furthermore, HSP47 contributes to angiogenesis 75 by activating extracellular signal-regulated kinase signaling and upregulating VEGF-A via hypoxia-inducible factor-1α (HIF-1α). These interconnected pathways highlight HSP47 as a central player in cancer development and a promising target for therapeutic intervention. HSP47 might actively promote angiogenesis in glioma through the HIF1α–VEGFR2 pathway. 75 In bladder cancer, the ERK signaling pathway, in conjunction with C–C motif chemokine ligand 2 (CCL2), fosters HSP47-mediated angiogenesis. 76 Studies indicate that HSP47 inhibitors can counteract VEGF-driven fibrovascular changes in retinal diseases, reducing the fibrovascular formation that contributes to conditions like retinal fibrosis. 77 This effect suggests a broader therapeutic potential for HSP47 inhibitors in controlling pathological angiogenesis beyond oncology.

### HSP47 Inhibition as a New Treatment for Thrombosis

Thrombosis contributes to approximately 25% of global mortality, underscoring its critical status as a leading public health threat. It is central to the pathophysiology of numerous cardiovascular complications, including myocardial infarction, stroke, and venous thromboembolism (VTE)—a condition encompassing deep vein thrombosis (DVT) and pulmonary embolism. 78 Risk factors for VTE range from classic contributors like cancer and surgery to immobilization alone, which independently elevates thrombotic risk even in otherwise healthy individuals. 79 The distinction between arterial and venous thrombosis is key: While platelets dominate arterial clotting through their adhesion to exposed collagen and subsequent aggregation, venous thrombosis primarily involves fibrin deposition, driven by coagulation factors and inflammatory mediators rather than platelets. Amidst the ongoing exploration of thrombogenic mechanisms, HSP47, traditionally known for its intracellular chaperoning of collagen, has emerged as a novel player in platelet biology. Proteomic analyses identified HSP47 on the surface of GPVI-activated platelets, challenging conventional views of its localization. 12 Functional studies revealed that HSP47 directly binds to collagen fibrils and facilitates platelet aggregation through collagen–GPVI interactions. Inhibiting HSP47 significantly diminished these interactions, highlighting its modulatory role in platelet–collagen adhesion. 13 80 Knockout mice lacking platelet-specific HSP47 or treated with HSP47 inhibitors displayed impaired thrombus formation under both in vivo and flow-based in vitro conditions, despite maintaining normal platelet counts. These mice also exhibited prolonged bleeding times, revealing HSP47's integral role in thrombus stability and hemostasis. 13

Interestingly, HSP47 functions not as a classical transmembrane receptor but more likely as a co-receptor or surface scaffold. 12 13 It enhances GPVI signaling pathways that lead to platelet adhesion and spreading, rather than initiating full activation independently. Studies demonstrate that collagen motifs bound by HSP47 support morphological changes like shape change, without inducing granule release or integrin αIIbβ3 activation. Moreover, HSP47 binds to the GFOGER motif—a recognized integrin α2β1 collagen-binding sequence—but does not directly modulate this interaction, 80 further supporting its role as an accessory regulator rather than a primary receptor.

Mechanistically, HSP47 is stored in the dense tubular system of resting platelets and translocates to the membrane upon activation via actin dynamics, similar to protein disulfide isomerase. 80 81 It enhances cytoskeletal remodeling, a trait observed in other cell types like fibroblasts and epithelial cells, 32 82 and is essential for promoting platelet spreading. It also stabilizes GPVI receptor dimerization and downstream phosphorylation of key signaling proteins such as spleen tyrosine kinase (Syk), linker for activation of T-cells (LAT), and phospholipase C gamma 2 (PLCγ2)—integral elements of the GPVI signaling axis. 80

Recent compelling data have further connected HSP47 to thrombin-mediated activation. Thienel et al. 14 demonstrated that HSP47 facilitates thrombin binding to platelets and is essential for downstream GPVI signaling, even though thrombin is traditionally associated with protease-activated receptor receptor activation. 14 Inhibition of HSP47 abrogated thrombin-triggered platelet aggregation, granule release, and neutrophil extracellular trap (NET) formation—events central to thromboinflammation. These findings position HSP47 as a nexus linking thrombin engagement to GPVI signal amplification. 14

In cancer-associated thrombosis (CT), HSP47 has also been implicated in platelet–cancer cell crosstalk through collagen secretion pathways, 71 reinforcing the protein's versatility beyond classical clot formation. Furthermore, studies in hibernating mammals such as brown bears reveal that their natural resistance to VTE during prolonged immobilization is associated with suppressed HSP47 levels, suggesting that downregulation of HSP47 may be an evolutionary thromboprotective adaptation. 14 Experimental models corroborate these observations. In PF4-Cre (+)/HSP47 ^−/−^ knockout mice subjected to inferior vena cava thrombosis, both thrombus size and frequency were significantly reduced. These mice also showed diminished NET formation and reduced circulating D-dimer and citH3/DNA complexes, further emphasizing HSP47's pivotal role in thromboinflammatory pathways. 14

Moreover, recent findings from whole exome sequencing of SERPINH1 have identified 99 variants, including missense and loss-of-function mutations. These variants were analyzed for differences in allele frequency between individuals with and without VTE. 83 The Malmö Diet and Cancer cohort, comprising 28,794 participants, was explored with a follow-up period extending until December 31, 2018. Among this population, 2,584 individuals experienced VTE events. Variants were assessed for their association with VTE using Cox regression models adjusted for age, sex, and additional risk factors such as body mass index (BMI), smoking, and Factor V Leiden. Kaplan–Meier survival analysis for thrombosis-free survival showed no significant differences in outcomes between carriers and non-carriers of SERPINH1 variants. Furthermore, genetic databases such as Genebass and UK Biobank exomes did not reveal significant links between SERPINH1 and thrombotic risk. Thus, the study concludes that SERPINH1 genetic variation is not associated with an increased risk of VTE in the general population, including both common and rare variants. While HSP47 has a critical biological role in platelet function and collagen interaction, its genetic variability does not appear to be a significant determinant of VTE risk in the studied population. 83

From a translational standpoint, this creates a compelling case for therapeutic targeting. Unlike conventional antiplatelet agents that often carry bleeding risks, HSP47 inhibition appears to offer antithrombotic benefits without significantly impairing primary hemostasis. Thienel et al. showed that small-molecule HSP47 inhibitors (SMI I and II) reduced thrombus formation in vivo without affecting bleeding time 14 —potentially overcoming one of the greatest limitations of current therapies like P2Y12 or GPIIb/IIIa inhibitors. Furthermore, recent studies indicate that HSP47 inhibitors reduce cerebral vascular damage in ischemic stroke models. 53 Structural work by Cai et al. 84 delineated the collagen-binding domains of HSP47 and identified potent inhibitory compounds capable of blocking its interaction with native collagens. These agents provide a rational basis for the development of selective, structure-guided inhibitors that target pathologic thrombosis without impairing physiological clotting mechanisms. Moreover, venous thrombosis is a common and severe complication in cancer patients, often driven by tumor-derived prothrombogenic extracellular vesicles (EVs). 85 86 87 Recent evidence, including data from Osorio et al., 88 highlights the presence of HSP47 on the surface of small EVs isolated from human blood. This is particularly relevant given HSP47's established roles in collagen stabilization and thrombosis via platelet–collagen interactions. Although HSP47 was traditionally viewed as an intracellular chaperone for collagen, its extracellular role—especially when bound to vesicular membranes—suggests a broader functional spectrum. The detection of HSP47 in small extracellular vesicles secreted in heart failure 88 supports the hypothesis that this chaperone may be externalized in pathological microenvironments such as those found in tumors. Since tumor hypoxia is a hallmark of aggressive malignancy and a known trigger for thromboinflammatory signaling, the presence of HSP47 in tumor-derived EVs may facilitate thrombin generation and platelet activation indirectly through interactions with collagen or other matrix components exposed at sites of vascular damage or inflammation. This aligns with prior findings where platelet-surface HSP47 supported GPVI signaling and promoted thrombus formation. Hence, in cancer-related thrombosis, tumor-derived EVs carrying HSP47 could mimic or enhance platelet–collagen interactions, thereby contributing to a procoagulant state. From a translational standpoint, this suggests that targeting vesicular HSP47—either directly through inhibitory antibodies or indirectly via blockade of EV uptake—may represent a novel antithrombotic strategy in oncology. Given the dual role of HSP47 in both fibrosis and thrombosis, its detection in EVs also opens avenues for biomarker development, helping to stratify cancer patients at elevated thrombotic risk. Finally, in regenerative medicine contexts, HSP47 appears to exert indirect influence on platelets through matrix remodeling. For instance, platelet-rich plasma and platelet-rich fibrin therapies have been shown to stimulate HSP47 expression in surrounding osteoblasts and tendon cells, promoting collagen synthesis and healing. 89 90 These findings suggest that activated platelets may influence extracellular HSP47 signaling in wound healing, even when HSP47 is not acting directly within the platelet itself.

Altogether, these multilayered insights into HSP47's role—spanning collagen binding, GPVI modulation, thrombin signaling, and matrix biology—support its candidacy as a highly promising therapeutic target in thrombotic diseases. Future efforts should aim to bridge preclinical discoveries with human applications, refine delivery mechanisms for selective inhibition, and delineate the contexts in which HSP47 modulation offers the greatest clinical value.

### HSP47 Inhibition: Is It Beneficial or Harmful?

The prospect of inhibiting HSP47 as a therapeutic strategy presents both opportunities and challenges, necessitating a nuanced approach to its application in medicine. HSP47, a collagen-specific molecular chaperone, plays a crucial role in ensuring proper collagen folding and stability. Its significance extends across various physiological processes, from maintaining structural integrity in bones to supporting ECM remodeling. Several compelling examples highlight the complexity of inhibiting HSP47 and emphasize the need for caution: The impact of HSP47 on bone integrity in OI, its potential necessity in hyperthermia (HT)-based cancer therapies, but also the potential impact on bleeding or autoimmune disorders.

First, OI is a genetic disorder characterized by fragile bones due to mutations affecting collagen production or structure, primarily type I collagen. OI Type X, a rare form of the disease, is caused by mutations in the *SERPINH1* gene, which encodes HSP47. 15 In this condition, defective HSP47 disrupts the proper folding and assembly of collagen, resulting in brittle bones, frequent fractures, and compromised skeletal integrity. Recent data have explored the role of the *SERPINH1* gene in another collagen disorder: hypermobile Ehlers–Danlos Syndrome (hEDS). The study investigates potential genetic contributions by sequencing the *SERPINH1* gene in 100 Polish patients diagnosed with hEDS. 91 Four heterozygous missense variants in the *SERPINH1* gene were identified in the study group. However, these variants were classified as benign. The study found no pathogenic or likely pathogenic mutations in *SERPINH1* , suggesting that the gene does not play a direct role in the etiology of hEDS. This result emphasizes the pivotal role of HSP47 in collagen biosynthesis and underscores the complexity of its potential involvement in various connective tissue diseases. The findings provide a foundation for further exploration into molecular mechanisms underlying hEDS and related disorders. Given its critical function, the inhibition of HSP47 could inadvertently mimic or exacerbate the pathological features of OI. Beyond bones, such inhibition may impair connective tissues throughout the body, leading to weakened tendons, poor wound healing, and vascular instability due to compromised collagen quality. Collagen is also vital for ECM remodeling, and disrupting this process could impair normal tissue repair and regeneration. To minimize these risks, therapeutic strategies targeting HSP47 must be carefully tailored, prioritizing selective inhibition in specific tissues or partial suppression to balance therapeutic benefits with essential physiological functions.

Second, HT, or thermal therapy, involves raising tissue temperatures to approximately 39 °C to 42 °C for about an hour. 92 93 94 95 This approach is widely studied in oncology, where it is often combined with chemotherapy, radiotherapy, or immunotherapy to enhance treatment efficacy. HT induces a range of beneficial cellular responses, including the upregulation of HSPs, such as HSP47. 96 97 This upregulation has been observed across species, including fish 98 99 and humans, where HSP47 is significantly elevated during thermal stress, aiding in collagen remodeling and tissue repair. In humans, several studies have demonstrated that HT induces an elevation in HSP47 mRNA transcription (Northern blot analysis) and protein translation (Western blot analysis) shortly after treatment. 100 101 102 103 104 105 This transcriptional and translational up-regulation is evident by the 6th hour, reaching its peak at the 9th hour. 106 Research in human dermal fibroblasts has also demonstrated that pulsed heat shocks at 60 °C result in greater collagen synthesis and reduced collagen degradation, leading to a higher overall net collagen content compared with heat shocks at 45 °C. 103 This stresses the importance of both temperature and pulse duration, as optimal pulses lasting approximately 2 seconds per rise significantly influence collagen remodeling and tissue repair. Additionally, these short-pulsed heat shocks at 45 °C and 60 °C led to an upregulation of both procollagen type I and procollagen type III. Studies examining HSP47 mRNA have also found a decrease in HSP47 transcription with aging. This phenomenon was mostly evident in cells from older individuals treated with HT, suggesting that the reduction in HSP47's heat response may contribute to the deteriorated quality and quantity of collagen observed with aging. 107 These examples highlight the dual nature of HSP47's role in medicine. Last but not least, HT shows promise in preventing thrombosis. 76 Indeed, in this recent study, the synergistic benefits of combining HT with intermittent pneumatic compression to enhance blood flow velocity and reduce the risk of DVT has been demonstrated. Temperatures between 42 °C and 45 °C were particularly effective, demonstrating superior hemodynamic outcomes while preserving vascular integrity. These findings suggest HT as a valuable intervention in thrombosis management following orthopedic procedures. 108

**Fig. 2:**
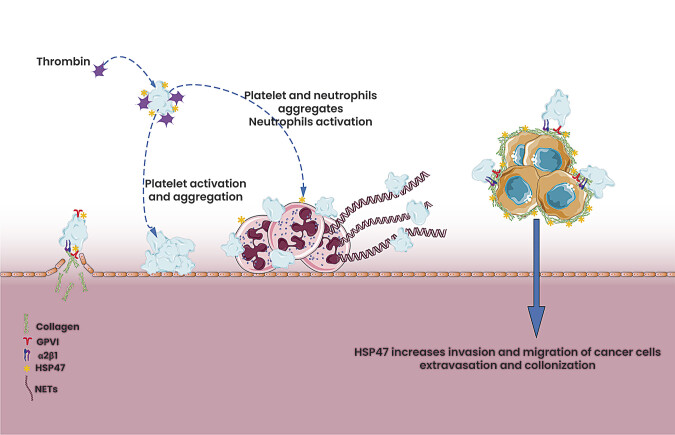
HSP47 orchestrates critical processes in platelet function and thromboinflammation. HSP47 facilitates thrombin recruitment and platelet aggregation while driving activation through GPVI-mediated signaling, enhancing thrombus formation. Beyond hemostasis, HSP47 activates polymorphonuclear leukocytes (PMNs), triggering the formation of neutrophil extracellular traps (NETs) composed of DNA and proteins, which provide a scaffold for thromboinflammation. Additionally, HSP47 strengthens platelet–cancer cell interactions, promoting tumor metastasis and contributing to a prothrombotic environment. HSP47, heat shock protein 47.

Thus, on one hand, HSP47 inhibition may offer therapeutic benefits in conditions like fibrosis or cancer. On the other hand, its essential role in maintaining collagen stability and tissue integrity, as evidenced by its inhibition in OI and its beneficial induction in HT-based therapies, highlights the potential risks associated with modulating HSP47. Moreover, the relationship between HT, thrombosis, and HSP47 regulation remains complex and somewhat controversial. The recent findings by Tong et al. underscore the dual nature of HT's effects. 108 While it effectively boosts blood flow and promotes venous return, it may also upregulate HSP47 could potentially contribute to thrombosis by promoting a procoagulant environment as described in Fig. 2 . These contrasting effects emphasize the need for cautious and context-specific therapeutic strategies. Modulating HSP47 requires a balanced approach to harness its benefits while mitigating potential risks.

Moreover, while our review has primarily centered on HSP47's role in thrombosis and fibrosis, its potential involvement in bleeding complications is worth further exploration. A study published in the “Journal of Thrombosis and Haemostasis” by Sasikumar highlights HSP47 as a platelet collagen-binding protein that contributes to thrombosis and hemostasis. 13 The study notes an increase in bleeding time associated with HSP47 activity, although there are currently no direct data available regarding its role in human cancer-related bleeding.

Finally, while targeting HSP47 has demonstrated promising therapeutic potential in fibrotic and thrombotic conditions, concerns arise regarding its inhibition in autoimmune diseases such as rheumatoid arthritis, where collagen integrity is already compromised. 109 110 Elevated HSP47 expression and the presence of anti-HSP47 autoantibodies are associated with autoimmune pathologies such as rheumatoid arthritis, systemic lupus erythematosus, and systemic sclerosis. 35 111 112 113 These findings suggest that inappropriate or non-selective inhibition of HSP47 may risk exacerbating inflammation or compromising tissue structural integrity in already vulnerable patients. Importantly, HSP47 is not only implicated in collagen maturation but also plays broader roles in angiogenesis, cellular stress responses, and immune modulation. It can be expressed aberrantly on the surface of activated cells and may act as a proinflammatory mediator in damaged tissues. This dual functionality underlines the necessity for strategies that achieve therapeutic inhibition of HSP47 while minimizing collateral effects on healthy tissue or immune tolerance. To address these risks, several innovative approaches are being developed. Tissue-specific delivery systems, such as antibody-guided or nanoparticle-based formulations, could allow targeted inhibition of HSP47 in fibrotic or thrombotic lesions while sparing tissues involved in joint integrity or immune regulation. Intermittent or low-dose administration may provide therapeutic effects during disease flares without compromising baseline collagen maintenance. Additionally, targeting HSP47 within the vasculature—particularly in endothelial cells or platelets implicated in thrombosis—may allow selective modulation of thromboinflammatory processes with a lower risk of systemic immune disruption. Furthermore, monitoring biomarkers such as circulating anti-HSP47 antibodies may help stratify patients according to risk and tailor dosing regimens appropriately. Such approaches, informed by the context-specific role of HSP47, would support the safe and effective development of HSP47-targeted therapies, especially in patients with coexisting autoimmune and fibrotic diseases.

## Conclusion

All in all, the discovery of HSP47's surface localization on platelets and its critical role in thrombosis represents one of the most extraordinary advancements in recent years within the thrombosis field. This revelation fundamentally alters our understanding of thrombus formation after immobilization, identifying HSP47 as a previously unrecognized but essential player in thrombosis. Its targeting presents an unparalleled opportunity for developing innovative antithrombotic therapies. Beyond thrombosis, HSP47's roles in fibrosis and cancer highlight its versatility and complexity. While it promotes collagen stability and tissue repair, its dysregulation can drive pathological processes such as fibrotic scarring and tumor progression. However, the relationship between HSP47 and HT introduces a nuanced perspective, particularly in oncology. This paradox suggests that inhibiting HSP47 in cancer may inadvertently undermine therapeutic strategies that rely on its beneficial effects during HT treatments. These dual roles demand careful modulation of HSP47 activity. While inhibition could alleviate conditions like fibrosis or thrombosis, it risks impairing essential collagen biosynthesis, as seen in OI Type X, or contradicting therapeutic gains in cancer HT protocols. This extraordinary discovery of HSP47's thrombogenic function underscores its centrality in disease biology. Harnessing this insight will not only advance therapeutic strategies for thrombosis but also provide a framework for addressing its roles in thrombosis, fibrosis, and cancer. Future research must prioritize tissue-specific and context-dependent modulation of HSP47 to unlock its full therapeutic potential while safeguarding its critical physiological functions. Overall, refining delivery strategies and therapeutic windows will be essential for translating HSP47 inhibition into a viable clinical intervention across diverse pathologies.
